# Quantitative analysis of macular contraction in idiopathic epiretinal membrane

**DOI:** 10.1186/1471-2415-14-51

**Published:** 2014-04-16

**Authors:** Jee Wook Kim, Kyung Seek Choi

**Affiliations:** 1 Aerospace Medicine Squadron, 18th Fighter Wing, Gangneung Air Base, Republic of Korea Air Force, Hak-dong, Gangneung-si, Gangwon-do, Korea; 2 Department of Ophthalmology, College of Medicine, Soonchunhyang University, 59 Daesagwan-ro, Yongsan-gu, Seoul 140-743, Korea

**Keywords:** Epiretinal membrane, ImageJ, Infrared fundus photography, Macular contraction, Macular thickness

## Abstract

**Background:**

We aimed to quantify the displacement of macular capillaries using infrared fundus photographs and image processing software (ImageJ) in patients with idiopathic epiretinal membrane (ERM) who have undergone vitrectomy and to analyze the correlation between vessel displacement and retinal thickness.

**Methods:**

This prospective study included 16 patients who underwent vitrectomy for idiopathic ERM. Ophthalmic examination and optical coherence tomography (OCT) were performed before and 3 months after surgery. The length of radial vessel segment included in each area (VLA) and the length from the foveola to the vessel branching point (FBL) depending on the superior, inferior, nasal, and temporal areas of the macula were measured using infrared fundus images and image processing software (ImageJ). Preoperative and postoperative parameters were compared and correlations between VLA, FBL, macular thickness, and visual acuity were assessed.

**Results:**

The VLA of superior, inferior, and temporal areas showed a significant postoperative reduction. VLA differences showed a positive correlation with differences in macular thickness, which corresponded to the superior, inferior, and temporal areas; however, no correlation was observed in the nasal area. The FBL of the superior and inferior areas was significantly increased postoperatively. A positive correlation was observed between FBL differences and macular thickness differences in the superior area. Postoperative change in VLA and FBL did not show a significant correlation with postoperative best corrected visual acuity (BCVA) and BCVA differences.

**Conclusions:**

Infrared fundus photographs and image processing software can be useful for quantifying progressive changes in retinal surface distortion after surgical removal of ERM. Macular edema and vascular distortion showed significant improvement after surgery. Furthermore, a correlation was observed between topographic and tomographic changes.

## Background

Epiretinal membrane (ERM) is a macular disease that induces blurred vision and metamorphopsia [1]. ERM has a fine and transparent appearance at early stage; however, as the membrane becomes thicker, the ERM causes the formation of radial wrinkles in the inner retina and changes the branching patterns of the retinal vessels [2-4]. Thus, contraction of retina caused by ERM may simultaneously induce retinal surface distortions and retinal edema.

Vitrectomy and removal of the membrane are recognized as the standard treatment for ERM; retinal surface distortions and retinal edema can be resolved after surgery [5,6]. Furthermore, changes in retinal thickness before and after surgery can be quantified using optical coherence tomography (OCT) [7,8]. However, a standardized method for quantifying retinal surface distortions has not yet been established. In addition, relatively few studies pertaining to the correlation between the natural course of ERM and the severity of macular contractions have been reported. Kofod et al. [9], who reported on measureable retinal vessel movement in ERM patients, described the correlation between retinal vessel movements, worsening of best corrected visual acuity (BCVA), and increased central macular thickness (CMT). In other studies, macular contraction was measured by comparing the displacement of the large blood vessel junction or an area under major vessels from consecutive fundus photographs or red-free photographs [6,10]. However, implementing these methods is difficult when the displacement of large vessels is limited. In addition, these methods cannot assess the fine movement of capillaries around the macula.

Accordingly, we conducted this study in order to directly quantify the displacement of macular capillaries using an infrared fundus photograph and image processing software on ERM patients who underwent vitrectomy. The secondary goal was to analyze the correlation between the amount of retinal vessel displacement and changes in retinal thickness after surgical removal of ERM, and to determine whether this change correlated with visual acuity improvement.

## Methods

### Subjects

This prospective study included 16 patients who underwent surgery for idiopathic ERM. All patients were observed for more than 3 months after surgery. Exclusion criteria were as follows: past history of ocular inflammation, retinal vascular disease, prior posterior segment surgery, and laser photocoagulation. Patients with a spherical equivalent over ± 6.0 diopters were also excluded, as measurement of retinal thickness may be affected in these subjects. This study followed the tenets of the Declaration of Helsinki and was approved by the institutional review board of Soonchunhyang University Hospital. Signed informed consent was obtained from all participating subjects.

### Surgical method and ophthalmic examination

All surgeries were performed by a single surgeon (K. S. Choi) at Soonchunhyang University Hospital. The surgical technique involved a standard 23-gauge pars plana vitrectomy and peeling of the ERM using indocyanine green dye, intraocular forceps, and a diamond dusted membrane scraper. In patients with cataracts, phacoemulsification and posterior chamber lens implantation were performed concurrently. BCVA and refractive errors were measured before and 3 months after surgery. Refraction was performed using a Snellen chart and measurements were recorded by trained optometrists. For statistical analysis, BCVA was converted to logarithm of the minimal angle of the resolution (logMAR).

### Change in retinal surface

Macular vessel displacements were recorded on fundus images obtained using The Spectralis® (Heidelberg Engineering, Heidelberg, Germany) Infrared + OCT setting. We used the standard 30-degree single line scan and a 30-degree infrared fundus photograph. Follow-up OCT scan was accurately superimposed on top of the preoperative OCT scan using the Spectralis AutoRescan modality. These aligned fundus photographs enable measurement of the same part of the retina over time.

Infrared fundus images were imported into a freeware image processing software (ImageJ) [11,12] and overlaid with a subfield grid. We employed the ‘modified’ Early Treatment Diabetic Retinopathy Study (ETDRS) macular grid. The ‘standard’ ETDRS grid is a 6 mm ring centered on the fovea. The inner subfields are bound by a 3 mm diameter ring, and the central subfield is bound by the innermost 1 mm ring. We simplified nine retinal subfields in the ‘standard’ ETDRS grid into five subfields: superior, inferior, temporal, nasal, and central subfield, and calculated the displacement of vessels within the individual subfields. The foveola was identified on one of the B-scans of volume OCT, and marked on the fundus photograph. The grid was manually placed on the fovea identified on infrared fundus photographs by a trained observer using ImageJ in the Department of Ophthalmology at Soonchunhyang University Hospital.

**Figure 1:**
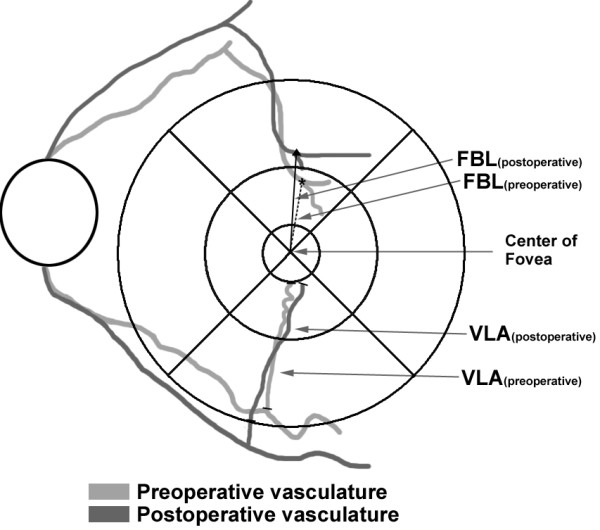
**A schematic showing the two features of change in the retinal surface.** (Upper quadrant) Length from the foveola to the vessel branching point (FBL) was calculated after selection of five points of the vessel branching points located at the superior and the inferior quadrant, in order of proximity to foveola. (Lower quadrant) Vessel segment length in area (VLA) was defined as the vessel segment length for radial vessels included in each quadrant consisting of a 1 mm and 6 mm diameter ring. Only vessel segments included between the 1 mm ring and the 6 mm ring was measured. The VLA was measured by selecting 3–5 radial arterioles or venules in each quadrant. FBL and VLA were calculated using infrared fundus photographs and image processing software (ImageJ).

Displacement of retinal vessels was observed by two parameters: the vessel segment length in area (VLA) and the length from the foveal center to the vessel branching point (FBL; Figure 1). We hypothesized that if the distortion of retinal vessels decreases after ERM surgery, the length of retinal vessel segment included in the specific space would shorten. VLA was defined as the vessel segment length for radial vessels included in each area. VLA extended from where the vessel crosses the 6 mm ring to where it crosses the 1 mm ring. In cases where the end of the capillary does not touch the 1 mm ring or the vessel branching point is located outside the 6 mm ring, only vessel segments included between the 1mm ring and 6mm ring were measured. The VLA was calculated semiautomatically from the infrared fundus photograph using ImageJ; 3–5 radial arterioles or venules in each quadrant were manually selected and values were averaged to a single value for each quadrant. Circumferential or oblique retinal vessels with a branching pattern that was not directed at the fovea were excluded.

**Figure 2:**
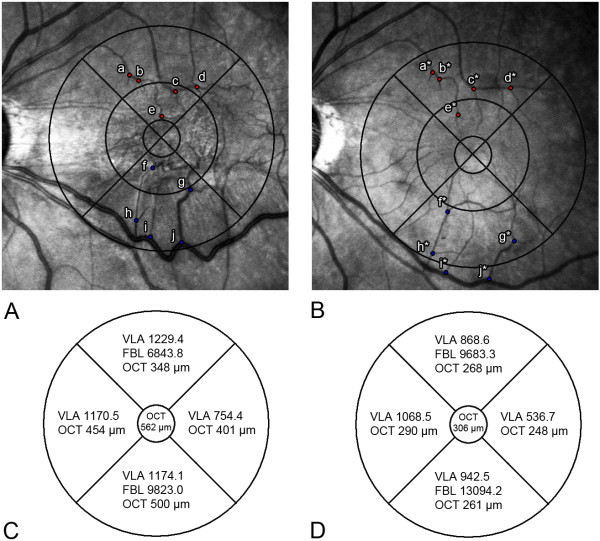
**Comparison of pre- and postoperative topographic parameters in the same patient with ERM. (A)** Infrared fundus photograph (overlaid with Early Treatment Diabetic Retinopathy Study [ETDRS] subfield) shows tortuous macular vessels preoperatively. Points a to j indicate the vessel branching points located at the superior and inferior areas in order of proximity to fovea **(B)** Postoperative infrared fundus photograph shows release of macular contraction. Points a* to j* indicate displaced branching points after surgery. **(C)** Actual preoperative measurement values of the vessel segment length in area (VLA), the length from the foveal center to the vessel branching point (FBL), and optical coherence tomography (OCT) are shown in each area. **(D)** Postoperative VLA, FBL, and OCT values are shown.

FBL was defined as the distance from the center of the fovea to the vessel branching point. In each of the superior and inferior quadrants, five recognizable retinal vessel branching points were selected in the order of proximity to the foveola. Distances from the foveola to each vessel branching points were calculated using ImageJ. We summed the five FBLs to generate a single value representing the total movement of branching points. The same branching points were selected in the follow-up photograph, and the distances were recorded. The mean value was used for analysis by repeated measurement performed three times each by different observers. The image observers were blinded to patient symptoms, BCVA, and macular thickness (MT) results. Preoperative and postoperative (3 months after surgery) values of the two parameters of retinal topographic features were compared (Figure 2). The authors also measured median and quartiles of the VLA and FBL values for evaluation of variability.

### Optical coherence tomography

MT was measured using a 30 × 25 degree volume scan with 61 sections using Spectralis® HRA + OCT (Heidelberg Engineering, Heidelberg, Germany). A 30 × 25 degree volume scan included the entire 6 mm ETDRS ring in all participants. MT corresponding to the ETDRS grid was measured in μm units using a retinal thickness map analysis protocol to display the numeric averages of the measurements for each area. The Spectralis AutoRescan modality utilizes active eye tracking, which provides a detailed retinal map and corrects for eye movement. The follow-up OCT image was superimposed on top of the baseline scan. The AutoRecan uses the same fundus imaging modality as recorded in the baseline scan [9].

### Statistical analysis

SPSS version 14.0 (IBM Corporation, Armonk, NY, USA) was used for statistical analyses. Wilcoxon signed rank test was used to compare preoperative and postoperative BCVA, VLA, FBL, and MT values. The Spearman correlation coefficient was used to examine correlations between ΔMT (preoperative MT – postoperative MT), ΔVLA (preoperative VLA – postoperative VLA), ΔFBL (postoperative FBL – preoperative VLA), and ΔBCVA (preoperative BCVA – postoperative BCVA).

## Results

A total of 16 subjects, nine males and seven females, were included in this study. The mean age of the patients was 63.6 ± 10.6 years (range 42–79 years). The mean spherical equivalent was – 0.39 ± 1.87 diopters. Preoperatively, 15 eyes were phakic and one eye was pseudophakic with a posterior chamber intraocular lens. All phakic eyes underwent combined cataract surgery with vitrectomy. Preoperative mean BCVA (logMAR ± standard deviation [SD]) was 0.31 ± 0.15 and preoperative central macular thickness (CMT) was 360.0 ± 102.6 μm (Table 1). Mean BCVA improved significantly 3 months postoperatively (0.08 ± 0.08; *P* = 0.001, Wilcoxon signed rank test), and postoperative CMT significantly reduced (302.5 ± 78.0 μm; *P* = 0.001).

**Table 1:** Demographic data of the patients

**Characteristics**	**Values**
Mean age (years)	63.6 ± 10.6 (42–79)
Male/female gender	9 (56.3%) / 7 (43.7%)
Refractive error (diopters)	−0.39 ± 1.87 (–3.75 to +3.50)
Phakic/pseudophakic (eyes, %)	15 (93.8%) / 1 (6.2%)
Preoperative BCVA (logMAR)	0.31 ± 0.15 (0.10–0.70)
Postoperative BCVA (logMAR)	0.08 ± 0.08 (0–0.20)
Preoperative central macular thickness (μm)	360.0 ± 102.6 (205–560)

BCVA, best-corrected visual acuity.

All values are given as mean ± standard deviation (range).

Changes in VLA were dependent on area; VLA of superior, inferior, and temporal areas was significantly reduced 3 months postoperatively (superior: *P* = 0.039; inferior: *P* = 0.013; temporal: *P* = 0.001), whereas no significant difference was observed in VLA of the nasal area (*P* = 0.082). FBL of the superior and inferior area was significantly increased 3 months postoperatively (superior: *P* = 0.006; inferior: *P* = 0.005). Significant changes in MT also depended on the specific area. MT of the superior, inferior, temporal and central areas was significantly reduced postoperatively (superior: *P* = 0.001; inferior: *P* = 0.05; temporal: *P* = 0.007; central: *P* = 0.001); however, no significant difference was observed in MT of the nasal area (*P* = 0.126; Table 2). Median and quartiles of VLA and FBL values are shown in Table 2.

**Table 2:** Comparison of preoperative and postoperative parameters of topographic features and macular thickness

**Parameters**		**Values**	**Median (q25–q75)**	***P*-value**
***Vessel segment length in area***				
*Preoperative*				
	*Superior*	1522.2 ± 233.5	1558.2 (1428.2–1622.2)	—
	*Inferior*	1401.6 ± 378.9	1363.0 (1250.5–1563.4)	—
	*Nasal*	1305.7 ± 250.2	1375.7 (1181.3–1435.2)	—
	*Temporal*	1515.6 ± 344.9	1529.6 (1358.8–1704.6)	—
*Postoperative*				
	*Superior*	1175.6 ± 239.3	1142.7 (1063.1–1371.5)	0.039^a^*
	*Inferior*	1043.5 ± 226.7	1095.4 (943.5–1155.6)	0.013^a^*
	*Nasal*	1049.0 ± 205.9	1069.5 (1026.5–1163.5)	0.082^a^
	*Temporal*	630.1 ± 179.0	654.1 (533.7–712.1)	0.001^a^*
***Length from the foveal center to the vessel branching point***				
*Preoperative*				
	*Superior*	9173.9 ± 1687.8	9339.4 (7837.4–10288.7)	—
	*Inferior*	9730.7 ± 1738.3	9880.2 (9669.0–10703.0)	—
*Postoperative*				
	*Superior*	9868.8 ± 1614.7	9819.5 (8669.2–10662.6)	0.006^a^*
	*Inferior*	10366.9 ± 1773.9	10628.3 (9661.0–11729.9)	0.005^a^*
***Macular thickness***				
*Preoperative*				
	*Superior*	340.5 ± 50.5	—	—
	*Inferior*	341.6 ± 78.5	—	—
	*Nasal*	375.5 ± 57.2	—	—
	*Temporal*	373.9 ± 64.9	—	—
	*Central*	368.3 ± 102.6	—	—
*Postoperative*				
	*Superior*	290.0 ± 41.2	—	0.001^a^*
	*Inferior*	279.0 ± 39.2	—	0.050^a^*
	*Nasal*	324.1 ± 41.3	—	0.126^a^
	*Temporal*	286.8 ± 52.5	—	0.007^a^*
	*Central*	302.5 ± 78.0	—	0.001^a^*

q25, lower 25% quartile; q75, upper 75% quartile.

All values are given as mean ± standard deviation.

^a^*P*-value by Wilcoxon signed-rank test between preoperative and postoperative values.

*P-value <0.05.

**Figure 3:**
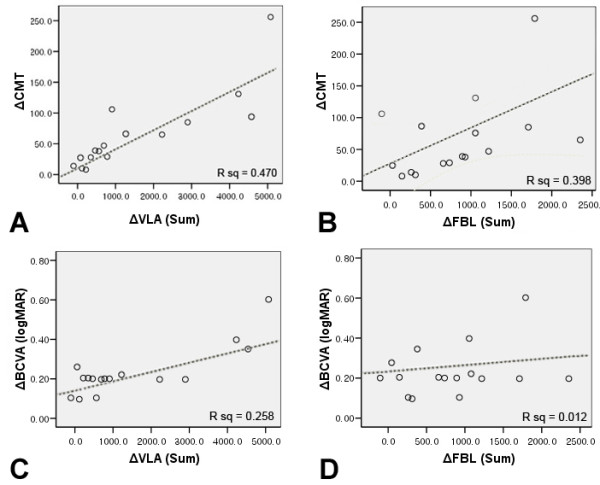
**Linear regression analysis of representative parameters.** Linear regression analysis of the vessel segment length in area (VLA), the length from the foveal center to the vessel branching point (FBL), central macular thickness (CMT), and best corrected visual acuity (BCVA). **(A)** Linear regression analysis shows a positive correlation between the sum of ΔVLA (preoperative VLA – postoperative VLA) and ΔCMT (preoperative CMT – postoperative CMT). **(B)** The sum of ΔFBL (postoperative FBL – preoperative VLA) shows a positive correlation with ΔCMT. **(C)** ΔBCVA (preoperative BCVA – postoperative BCVA) does not show a significant correlation with the sum of ΔVLA. **(D)** ΔBCVA does not show significant correlation with the sum of ΔFBL.

ΔVLA showed a positive correlation with ΔMT, which corresponded to superior, inferior, and temporal areas (superior: r = 0.813, *P* = 0.001; inferior: r = 0.593, *P* = 0.033; temporal: r = 0.872, *P* = 0.000; Spearman correlation coefficient); however, in the nasal area, no correlation was observed between ΔVLA and ΔMT (nasal: r = 0.632, *P* = 0.521). In addition, the sum of ΔVLA in all areas showed a positive correlation with ΔCMT (preoperative CMT – postoperative CMT) (r = 0.687, *P* = 0.010; Table 3; Figure 3).

**Table 3:** Correlation of differences in topographic features and macular thickness

**Parameters**	**Values**	**r**	***P*-value**
**Δ vessel segment length in area**			
Superior	346.6 ± 269.0	0.813	0.001*
Inferior	358.1 ± 605.0	0.593	0.033*
Nasal	256.7 ± 261.4	0.632	0.521
Temporal	885.5 ± 656.6	0.872	<0.001*
Sum	1846.9 ± 1556.8	0.687	0.010†
**Δ length from the foveal center to the vessel branching point**			
Superior	694.9 ± 633.8	0.560	0.046*
Inferior	636.2 ± 496.3	0.505	0.059
Sum	1331.2 ± 763.2	0.610	0.027†
**Δ macular thickness**			
Superior	50.5 ± 41.5		—
Inferior	62.6 ± 132.3		—
Nasal	51.4 ± 87.2		—
Temporal	87.1 ± 66.6		—
Central	65.8 ± 68.4		—

All values are given as mean ± standard deviation (μm).

*P-value by Spearman correlation between Δlength and Δmacular thickness of each area.

†*P*-value by Spearman correlation between Δlength and Δcentral macular thickness.

ΔFBL showed a positive correlation with ΔMT in the superior area (r = 0.560; *P* = 0.046) and a borderline correlation with ΔMT was observed in the inferior area (r = 0.505; *P* = 0.059). In addition, the sum of ΔFBL showed a positive correlation with ΔCMT in all areas (r = 0.610, *P* = 0.027; Table 3; Figure 3).

Regarding visual acuity parameters, ΔVLA and ΔFBL showed a positive correlation with preoperative BCVA (r = 0.704, *P* = 0.007 and r = 0.730, *P* = 0.005, respectively); however, postoperative BCVA and ΔVLA (*P* = 0.661), postoperative BCVA and ΔFBL (*P* = 0.057), ΔBCVA and ΔVLA (*P* = 0.102), and ΔBCVA and ΔFBL (*P* = 0 .800) did not show a significant correlation (Figure 3).

## Discussion

The main objective of this study was to quantify macular contraction using infrared fundus images and ImageJ from patients who underwent ERM surgery. The current work demonstrates that objective calculation and visualization of progressive retinal surface distortion changes after surgical removal of ERM can be obtained using this method. The postoperative decreases in VLA and increases in FBL after ERM surgery may be associated with retinal distortion relief. Compared with previous studies, which indirectly assumed retinal surface changes by measuring displacement of large retinal vessels and changes in capillaries around the fovea were difficult to assess due to the membrane, our method has the advantage of enabling direct measurements of retinal capillary movement around the fovea [6,10]. In particular, compared with other studies in which only overall changes pertaining to macular contraction were detected, VLA was used to evaluate whether there was a difference in capillary movement and macular contraction in specific areas of the macula [6,10]. Furthermore, FBL indirectly assessed retinal surface changes by measuring displacement of vessel branching points similar to previous studies; however, our method is simpler and easier to apply [6,10].

A change in retinal surface vessel distribution was observed using two parameters: ΔVLA and ΔFBL. The sum of ΔVLA and ΔFBL showed a positive correlation with ΔCMT in all areas, indicating that the amount of horizontal and vertical change of the macula are related [6]. We measured VLA by selecting 3–5 radial retinal vessels included in each area. Circumferential or oblique retinal vessels that were not directed at the fovea were excluded. In practice, because measurement of the length of all vessels in each area is difficult, the result could be affected by which vessels were selected. This may be a limitation of our study. However, three repeated measurements performed by different observers and the use of mean value for analysis could render our results applicable.

In this study, a difference was observed in VLA change depending on the area. VLA of superior, inferior, and temporal areas was significantly reduced after surgery; by contrast, no significant difference was observed in VLA of the nasal area. In a previous study conducted in unilateral ERM patients, preoperative optic disc-to-fovea distance was not significantly different from that of the unaffected eye, indicating little change in disc-to-fovea distance by retinal contraction due to ERM [6]. Due to the fixed location of the optic disc, only a small contraction was induced by ERM in the nasal area; therefore, no significant difference in VLA of the nasal area was observed after ERM removal.

The relationship between anatomical and functional recovery after ERM removal is controversial [13-15]. In this study, significant improvement in visual acuity and macular thickness was observed after surgery. However, ΔVLA, ΔFBL, and ΔCMT were not related to postoperative BCVA and ΔBCVA. Therefore, this change may be associated with the effect of combined cataract surgery. In a previous study, improvement in visual acuity after ERM surgery was greater in patients who underwent combined cataract surgery [16]. In our study, all patients with a phakic eye underwent combined cataract surgery, and there was no occurrence of after-cataract in all cases during the follow-up period. Therefore, the grade of cataract and the effect of cataract surgery on visual improvement of each patient may serve as a confounding factor in the relationship between anatomical restoration and improvement of visual acuity. In addition, anatomical recovery generally precedes improvement in visual acuity [17]. Although most patients are known to have restored functional improvement 1 or 2 months after ERM surgery, improvement in visual acuity has been reported years after surgery [4]. In our study, a relatively short follow-up period might have influenced the correlation between anatomical and functional improvement.

According to previous reports, unrecovered metamorphopsia and contrast sensitivity, despite anatomical recovery, may influence the evaluation of subjective visual recovery [1,18,19]. However, in this study, we did not include subjective functional improvement, such as metamorphopsia and contrast sensitivity other than visual acuity. In addition, inclusion of surgically treated ERM may induce a selection bias in that more severe forms of ERM were selected and ERM that does not contract was excluded. Therefore, long-term studies using patients with ERM of varying severity and including subjective functional improvement (such as metamorphopsia or contrast sensitivity) are warranted.

## Conclusions

In conclusion, using infrared fundus images and image processing software can be clinically useful for quantifying progressive changes in retinal surface distortion after surgical removal of ERM and has the advantage of measuring the movement of retinal capillaries around the fovea. Macular edema and vascular distortion significantly improved after surgical removal of ERM, and correlation was observed between topographic and tomographic changes.

## Competing interests

The authors declare that they have no competing interests.

## Authors’ contributions

Both of the authors contributed substantially to this study, including the analysis and interpretation of clinical data, the development and writing of the manuscript, and approved the final draft for publication.

## Pre-publication history

The pre-publication history for this paper can be accessed here:

http://www.biomedcentral.com/1471-2415/14/51/prepub
